# Outcomes of an Intravenous to Subcutaneous Infliximab (CT‐P13) Strategy in Takayasu Arteritis: A Proof‐of‐Concept Prospective Study

**DOI:** 10.1002/acr2.70158

**Published:** 2026-01-11

**Authors:** Luca Iorio, Federica Davanzo, Eleonora Fiorin, Marta Codirenzi, Roberta Prevedello, Andrea Doria, Roberto Padoan

**Affiliations:** ^1^ Rheumatology Unit, Department of Medicine University of Padua Padua Italy

## Abstract

**Objectives:**

To evaluate persistence, outcomes, safety, and remission maintenance after switching from intravenous infliximab (IV‐IFX) to subcutaneous infliximab (SC‐IFX, CT‐P13) in patients with Takayasu arteritis (TA).

**Methods:**

We conducted a prospective, single‐center, proof‐of‐concept observational study of consecutive adults with TA in sustained clinical, laboratory, and positron emission tomography (PET) remission switched from stable 5 mg/kg dose IV‐IFX to SC‐IFX 120 mg every two weeks. Assessments at baseline, 6 months, and 12 months included Indian Takayasu Clinical Activity Score (ITAS2010), Disease Extent Index‐Takayasu (Dei.TAK), PET Vascular Activity Score (PETVAS), C‐reactive protein (CRP) level, erythrocyte sedimentation rate (ESR), Large Vessel Vasculitis Index of Damage (LVVID), and safety data. The primary objective was 12‐month drug persistence. Secondary outcomes included remission rates, changes in ITAS2010, Dei.TAK, PETVAS, acute‐phase reactants, damage accrual, and safety.

**Results:**

Nine women (median age 35 [interquartile range (IQR) 28–44] years) were included. The median IV‐IFX exposure before the switch was 60 (IQR 24–72) months. Twelve‐month drug persistence was 77.8%. One patient discontinued early due to an injection‐site reaction, and another experienced a relapse at 12 months that required a therapy change. At 12 months, 87.5% (seven of eight) patients remained in remission. CRP and ESR remained stable (*P* = 0.297 and *P* = 0.068, respectively). PETVAS was similar to pre‐switch values (*P* = 0.589). Median LVVID remained stable throughout follow‐up. No serious adverse events occurred.

**Conclusions:**

IV‐IFX to SC‐IFX switching was feasible and well tolerated and generally maintained remission over 12 months in most patients with TA, with stable metabolic imaging and no increase in damage accrual. These findings support SC‐IFX as a practical maintenance strategy, warranting confirmation in larger multicenter cohorts.

## INTRODUCTION

Takayasu arteritis (TA) is a rare granulomatous large‐vessel vasculitis that primarily affects the aorta and its major branches. It predominantly affects young women and is associated with significant morbidity and premature mortality.^1^, ^2^, ^3^ Treatment options include glucocorticoids (GCs), conventional disease‐modifying antirheumatic drugs (cDMARDs), biologic DMARDs (bDMARDs), and surgical or endovascular treatment of vascular abnormalities. Among bDMARDs for refractory or relapsing TA, both tumor necrosis factor α inhibitors (TNFi) and interleukin‐6 receptor antagonists have become integral treatment options and are included in recent international and national guidelines.^2^, ^4^ Current evidence does not clearly favor TNFi over tocilizumab; therefore, treatment choice should be individualized based on prior response, disease phenotype, and tolerability profiles.^2^, ^4^

**SIGNIFICANCE & INNOVATIONS**

- In this prospective real‐world study, switching stable patients with Takayasu arteritis (TA) from intravenous to subcutaneous infliximab is feasible, with most patients maintaining remission.
- Stability across clinical scores, acute‐phase reactants, and positron emission tomography, coupled with no progression of damage over 12 months, offers coherent evidence that remission can be sustained after the switch.
- Treatment was well tolerated, with only minor events, supporting the acceptability of subcutaneous formulation and laying groundwork for broader maintenance strategies in TA.

Infliximab (IFX), a chimeric anti‐TNFi monoclonal antibody, is among the agents with the strongest evidence for inducing and sustaining remission in TA.^5^, ^6^ A subcutaneous formulation of biosimilar IFX (CT‐P13) has recently become available for rheumatoid arthritis, spondyloarthritis, and inflammatory bowel diseases. Compared with intravenous (IV) IFX, the subcutaneous (SC) route offers practical advantages, including a more stable pharmacokinetic profile, potentially reduced immunogenicity, decreased use of hospital resources, and improved patient convenience and health‐related quality of life through self‐administration and time savings.^7^, ^8^, ^9^ No evidence specific to TA exists, particularly on the effectiveness and safety of switching stable patients from IV‐IFX to SC‐IFX.

This prospective study aimed to evaluate drug persistence, clinical outcomes, safety, and the ability to maintain remission after switching from IV‐IFX to SC‐IFX in patients with TA.

## METHODS

### Patient population and outcomes

We conducted a prospective, single‐center, proof‐of‐concept observational study of consecutive adult patients with TA observed at the Padua Vasculitis Center between January 2023 and July 2024. All patients fulfilled the 2022 American College of Rheumatology (ACR)/EULAR classification criteria for TA.^1^, ^3^ Eligible patients were aged ≥18 years and in sustained remission at switch, defined by (1) the absence of TA‐related signs or symptoms, (2) normal acute‐phase reactants, and (3) no abnormal vascular metabolic activity on 18‐fluorodeoxyglucose positron emission tomography (^18^F‐FDG‐PET). Patients had received IV‐IFX at a stable dose of 5 mg/kg every four to eight weeks^4^ and were switched to SC‐IFX 120 mg every two weeks. Concomitant therapies (GCs and cDMARDs) were required to be stable for four or more weeks before switch; GC tapering/discontinuation after switch was allowed per clinician judgment. To minimize selection bias, all consecutive patients with TA who fulfilled the inclusion criteria during the study period were enrolled.

The primary outcome was 12‐month drug persistence on SC‐IFX, defined as time from switch to permanent discontinuation (switch to another biologic/targeted therapy, investigator‐declared stop, or dosing gap greater than or equal to eight weeks beyond schedule); patients without discontinuation were censored at last follow‐up. Secondary outcomes were (1) clinical remission at 12 months, (2) changes in disease activity scores as well as C‐reactive protein (CRP) level and erythrocyte sedimentation rate (ESR) at 6 and 12 months, (3) safety profile, and (4) damage accrual. The deidentified data of the case series are available on request from the corresponding author.

### Data collection and assessment

Clinical, laboratory, treatment, and outcome data were prospectively collected at baseline (switch), after 6 and 12 months, and at last available follow‐up. Disease activity was assessed with the Indian Takayasu Clinical Activity Score (ITAS2010),^10^ the Disease Extent Index‐Takayasu (Dei.TAK),^11^ and acute‐phase reactants.^12^ The vascular metabolic activity on PET was quantified with the PET Vascular Activity Score (PETVAS).^13^ All PET scans were interpreted by an experienced nuclear medicine physician blinded to clinical data and treatment phase, in accordance with standard clinical practice. Damage accrual was measured using the Large Vessel Vasculitis Index of Damage (LVVID),^14^ which records complications such as ischemic optic neuropathy, arterial occlusions/critical stenosis, aneurysms, and damage requiring surgery/intervention. To complete the score, structural vascular imaging was performed according to clinical indication. Safety was captured through chart‐recorded adverse events and routine laboratory monitoring. Severe adverse events included death, life‐threatening events, irreversible organ damage, or hospitalization. Other adverse reactions were classified as mild adverse events.

### Statistical analysis

Categorical variables are presented as frequencies and percentages, while continuous variables are presented as medians with interquartile ranges (IQRs). The normality of data distribution was assessed using the Shapiro–Wilk test. Nonparametric methods were used since normality was not met.

Treatment persistence was estimated by Kaplan–Meier with 95% confidence intervals (CIs) and censoring at last follow‐up. Changes over time in continuous outcomes were analyzed with the Friedman test for repeated measures (baseline, 6 months, and 12 months), followed by Wilcoxon signed rank tests for pairwise comparisons with Bonferroni adjustment, with *P* values ≤0.003 considered statistically significant. Categorical variables were compared using the chi‐square test or Fisher's exact test, as appropriate, with *P* values ≤ 0.003 considered statistically significant according to the Bonferroni correction for multiple comparisons. The statistical analyses were performed using IBM SPSS Statistics, version 20.0 (IBM Corp) and the free software Jamovi (version 2.4.11).

### Ethics approval

The study was approved by the local Ethics Committee “Comitato Etico Territoriale Area Centro – Est Veneto” (protocol n AOP3821, CET‐ACEV 6329/EST/25). Informed consent was obtained from the patients. All patients were informed that their clinical data could be used anonymously for research and gave their consent for the use of their data unless they provided an opposition to it, which none of the patients provided.

## RESULTS

A total of nine consecutive patients with TA were switched to SC‐IFX and included. All patients were women (9/9, 100%). According to Numano angiographic classification,^15^ six (66.7%) patients had type I, two (22.2%) patients had type IIb, and one (11.1%) patient had type V. Table 1 summarizes demographics and clinical characteristics of the cohort at diagnosis.

**Table 1:** Patient demographics and clinical characteristics at diagnosis*

	Patients with Takayasu arteritis (n = 9)
Demographics	
Female, n (%)	9 (100)
Age at disease onset, median (IQR), y	25 (18–29)
Age at diagnosis, median (IQR), y	27 (24–32)
2022 ACR/EULAR classification criteria, n (%)	8 (88.9)
Clinical manifestations at diagnosis, n (%)	
Headache	6 (66.7)
Fatigue	6 (66.7)
Vascular bruits	6 (66.7)
Pulse loss	4 (44.4)
Pulse/blood pressure inequality	4 (44.4)
Fever	4 (44.4)
Arthritis/arthralgia	3 (33.3)
Ocular involvement ‐ blurring vision	3 (33.3)
Carotidynia	2 (22.2)
Weight loss	1 (11.1)
Syncope	1 (11.1)
Dizziness or vertigo	1 (11.1)
Ocular involvement ‐ inflammatory ocular disease	1 (11.1)
Limbs claudication	1 (11.1)
New onset arterial hypertension	1 (11.1)
Chest pain	1 (11.1)
Erythema nodosum	1 (11.1)
TIA or stroke, seizure, acute kidney failure, abdominal angina, and bowel infarction	0 (0)
Numano angiographic classification, n (%)	
Type I	6 (66.7)
Type IIa	0 (0)
Type IIb	2 (22.2)
Type III	0 (0)
Type IV	0 (0)
Type V	1 (11.1)
Number of arteries affected, n (%)	
One artery	2 (22.2)
Two arteries	4 (44.4)
≥Three arteries	3 (33.3)

* Continuous variables are expressed by median and IQR, whereas categorical variables are expressed by frequencies and percentages. ACR, American College of Rheumatology; IQR, interquartile range; TIA, transient ischemic attack.

The median age at IFX switch was 35 (IQR 28–44) years, and disease duration was 7 (IQR 3–11) years; median BMI was 22.7 (IQR 20.2–25.2). Disease activity and markers of inflammation were low (CRP 1.4 [IQR 0.4–2.7] mg/L, ESR 13 [IQR 5–19] mm/hr, ITAS2010 0 [IQR 0–0], and Dei.TAK 0 [IQR 0–0]). The IV‐IFX treatment exposure before switch was 60 (IQR 24–72) months. No patient was receiving GCs. Three (33.3%) patients were taking concomitant cDMARDs (azathioprine, n = 2; methotrexate, n = 1). Before switching to SC‐IFX, six (66.7 %) patients had failed at least one cDMARD, two (22.2%) had failed at least two cDMARDs, and one (11.1%) had failed a prior biologic (tocilizumab). Over the preceding disease course, two patients had one relapse, two had two relapses, and one had three relapses. Table 2 reports detailed clinical and treatment features at switch.

**Table 2:** Patient demographics and clinical characteristics at switch*

Patient	Patient history from diagnosis to switch						Patient characteristics at switch							
	Numano angiographic classification	GCs therapy	DMARDs except for IFX	IV‐IFX therapy duration, mo	IV‐IFX treatment regimen (5 mg/kg)	Relapses	Age at switch, y	Disease duration, y	BMI	Current cDMARDs	CRP (mg/L)/ESR (mm/hr)	ITAS2010	Dei.TAK	LVVID
1	I	Yes	–	32	300 mg/6 wk	0	33	3	20.2	–	0.4/5	0	0	1
2	IIb	Yes	AZA	22	300 mg/4 wk	0	41	3	19.5	AZA	0.1/2	0	0	1
3	IIb	Yes	MTX	132	250 mg/5 wk	2	26	12	22.7	–	0.3/13	0	0	0
4	I	Yes	AZA, MTX, CsA, TCZ	60	400 mg/8 wk	2	35	8	25.2	–	1.4/19	0	0	1
5	I	Yes	MTX	72	300 mg/5 wk	1	61	7	21.0	–	0.6/31	0	0	1
6	I	Yes	AZA	24	300 mg/4 wk	0	20	5	18.6	–	2.9/5	0	0	1
7	V	Yes	MTX	24	300 mg/4 wk	0	28	2	28.4	MTX	1.9/18	0	0	1
8	I	Yes	CYC	60	300 mg/4 wk	3	55	30	34.8	–	2.7/30	0	0	2
9	I	Yes	AZA, MTX	130	300 mg/5 wk	1	44	11	24.4	AZA	2.9/5	0	0	2

* All pre‐switch IV infliximab doses were weight based at 5 mg/kg; absolute milligram values vary with body weight, and infusion intervals varied from every four to eight weeks. Post‐switch dosing was fixed at 120 mg every two weeks. AZA, azathioprine; BMI, body mass index; cDMARDs, conventional disease‐modifying antirheumatic drugs; CRP, C‐reactive protein; CsA, cyclosporine; CYC, cyclophosphamide; Dei.TAK, Disease Extent Index‐Takayasu; DMARDs, disease‐modifying antirheumatic drugs; ESR, erythrocyte sedimentation rate; GCs, glucocorticoids; IFX, infliximab; ITAS2010, Indian Takayasu Clinical Activity Score; IV, intravenous; LVVID, Large Vessel Vasculitis Index of Damage; MTX, methotrexate; PETVAS, positron emission tomography vasculitis activity score; TCZ, tocilizumab.

Twelve‐month drug persistence on SC‐IFX was 77.8% (7/9; 95% CI 36%–94%); two permanent discontinuations occurred. One patient (Table 2, patient 4) discontinued SC‐IFX after two months due to a local injection‐site reaction, remained off therapy, and relapsed six months later. The other patient (Table 2, patient 6) experienced a clinical and metabolic relapse (ITAS2010 = 2, Dei.TAK = 3, CRP = 60.5 mg/L, ESR 96 mm/hr, and a PETVAS score of 9) at 12 months and was switched to high‐dose oral GCs and tocilizumab.

At 12 months, seven of eight patients (87.5%) remained in clinical remission, defined by physician assessment, ITAS2010, and Dei.TAK. There were no significant changes from baseline to 6 and 12 months in acute‐phase reactants (CRP, *P* = 0.297; ESR, *P* = 0.068). All patients underwent a ^18^F‐FDG‐PET scan after at least 12 months of SC‐IFX therapy. PETVAS did not differ significantly from pre‐switch values (median 3 [IQR 0–5] pre‐switch vs 1 [IQR 0–2.3] after 12 months; *P* = 0.589). Damage accrual did not increase during the treatment, with a median LVVID of 1 (IQR 1.00–2.00) at 12 months. One patient was able to discontinue azathioprine during follow‐up. Safety was favorable: no severe adverse events or hepatotoxicity was recorded. No patient died during the observational period. Aside from the single injection‐site reaction leading to early discontinuation, only one nonsevere respiratory tract infection occurred. Safety and longitudinal outcomes are detailed in Table 3.

**Table 3:** Longitudinal changes in acute‐phase reactants, disease activity, prednisone use, and safety outcome over the follow‐up*

	Baseline (n = 9)	6 months (n = 8)	*P* value (T6 vs T0)	12 months (n = 8)	*P* value (T12 vs T0)
Clinical activity	0 (0)	0 (0)	1.000	1 (12.5)	0.274
CRP, mg/L	1.4 (0.4–2.7)	0.49 (0.29–0.83)	0.675	1.6 (0.6–2.5)	0.297
ESR, mm/hr	13 (5–19)	14.5 (5–27)	0.916	22.5 (14–46)	0.068
LVVID	1 (1–1)	1 (1–1.3)	–	1 (1–1.3)	1.000
ITAS2010	0 (0–0)	0 (0–0)	1.000	0 (0–0.3)	0.371
ITAS2010 = 0	9 (100)	8 (100)	1.000	6 (75)	0.110
Dei.TAK	0 (0–0)	0 (0–0.0)	1.000	0 (0–0.3)	0.371
Dei.TAK = 0	9 (100)	8 (100)	1.000	6 (75)	0.110
Patients taking GCs	0 (0)	0 (0)	1.000	1 (12.5)	0.274
Patients taking cDMARDs	3 (33.3)	3 (37.5)	0.857	2 (25)	0.707
Adverse events	0 (0)	0 (0)	1.000	0 (0)	1.000
Infections	0 (0)	2 (37.5)	0.110	2 (37.5)	0.110

* Data are expressed as frequency (%) and median (interquartile range). Each follow‐up timepoint is compared with the baseline. Categorical variables were compared using the chi‐square test or Fisher's exact test with Bonferroni correction for multiple comparisons (*P* values ≤ 0.003 considered statistically significant); continuous variables were analyzed using the Friedman test, followed by post hoc analysis with Wilcoxon signed rank tests with a Bonferroni correction applied for multiple testing (*P* values ≤ 0.003 considered statistically significant). cDMARDs, conventional disease‐modifying antirheumatic drugs; CRP, C‐reactive protein; Dei.TAK, Disease Extent Index‐Takayasu; ESR, erythrocyte sedimentation rate; GCs, glucocorticoids; ITAS2010, Indian Takayasu Clinical Activity Score; LVVID, Large Vessel Vasculitis Index of Damage; PETVAS, positron emission tomography vasculitis activity score.

## DISCUSSION

The availability of SC‐IFX across multiple immune‐mediated diseases is expanding and offers a meaningful advance for patients with TA, in which long‐term maintenance strategies remain challenging.^4^, ^9^ In this prospective single‐center cohort of patients with TA switched from IV‐IFX to SC‐IFX while in remission, the strategy was feasible, well tolerated, and effective in maintaining disease control in most cases. At one year, persistence was 77.8% and seven of eight evaluable patients remained in clinical remission, with no meaningful change in acute‐phase reactants. Notably, concordant stability was observed across laboratory and imaging domains. ^18^F‐FDG‐PET demonstrated no new vascular metabolic activity in the majority of patients, and PETVAS remained similar to pre‐switch values, reinforcing the impression of sustained disease quiescence after transition to SC‐IFX. Given the recognized difficulty of detecting subclinical activity in TA,^13^ this triangulation supports the ability of SC‐IFX to sustain remission after an IV to SC switch in appropriately selected patients. There was no progression of disease damage over one year, as reflected by stable LVVID scores, further arguing against silent accrual of large‐vessel damage in the short term. Finally, no significant safety concerns and intolerance were reported, with the exception of one patient who exhibited a local skin reaction. Notably, the only relapse at 12 months occurred in a patient previously treated with IV‐IFX every four weeks; given the sample size and single event, no causal inference can be drawn. Nevertheless, replacing individualized, weight‐based IV dosing with a fixed 120 mg every two weeks SC regimen may yield relative under‐ or overdosing at the individual level, underscoring the need for pharmacokinetic‐informed dose‐equivalence studies.

Our findings are consistent with emerging experience with SC‐IFX in rheumatoid arthritis, spondyloarthritis, and inflammatory bowel disease, where a more stable pharmacokinetic profile and potentially lower immunogenicity relative to IV formulations have translated into comparable or superior disease control.^7^, ^8^, ^9^ Our exploratory findings suggest no clear signal of loss of disease control after switching from IV‐IFX to SC‐IFX in patients in remission, but definitive conclusions will require prospective comparative studies.

Beyond effectiveness and safety, the shift to self‐administered SC therapy has pragmatic advantages for patients and health systems. It reduces reliance on infusion services, optimizes health care resources, and ensures greater convenience for individuals in their working and reproductive years, which may improve treatment acceptability and indirectly support long‐term adherence. These aspects are particularly relevant in TA, a disease that often affects young women who face cumulative treatment burdens over time.^7^ Our findings further support the feasibility of IFX in patients with TA, complementing existing experience with tocilizumab, and highlight the need for head‐to‐head comparative studies.^2^

Our study has some limitations. The small sample size and single‐center setting restrict inference and generalizability, and the lack of IV‐IFX comparator precludes direct conclusions about noninferiority. Immunogenicity and pharmacokinetic data were not collected, limiting mechanistic interpretation. Finally, the study was not powered to detect uncommon adverse events. Moreover, although all patients received a standardized switch protocol, dosing intervals and prior treatment intensity varied across individuals. For these reasons, the results should be interpreted as preliminary, proof‐of‐concept evidence that the IV to SC transition may be feasible and effective in TA. Nevertheless, strengths include a prospective design with uniform switch protocol; predefined remission criteria; systematic capture of clinical, laboratory, and PET‐based metabolic outcomes at 12 months; and formal assessment of damage accrual with LVVID. Taken together, these features provide coherent, multidomain evidence of maintenance of remission after switching to SC‐IFX, while underscoring the need for larger, multicenter studies to confirm and expand these findings.

In this real‐world prospective study, switching from IV‐IFX to SC‐IFX 120 mg every two weeks was feasible, well tolerated, and maintained disease remission in most patients with TA over 12 months, with stable metabolic imaging and no evidence of damage progression. These exploratory data support SC‐IFX as a practical maintenance option for appropriately selected patients and justify confirmation in larger multicenter studies.

## AUTHOR CONTRIBUTIONS

All authors contributed to at least one of the following manuscript preparation roles: conceptualization AND/OR methodology, software, investigation, formal analysis, data curation, visualization, and validation AND drafting or reviewing/editing the final draft. As the corresponding author, Dr Padoan confirms that all authors have provided the final approval of the version to be published, and takes responsibility for the affirmations regarding article submission (eg, not under consideration by another journal), the integrity of the data presented, and the statements regarding compliance with institutional review board/Declaration of Helsinki requirements.

## Supporting information


**Disclosure Form**:
